# High-field plasma acceleration in a high-ionization-potential gas

**DOI:** 10.1038/ncomms11898

**Published:** 2016-06-17

**Authors:** S. Corde, E. Adli, J. M. Allen, W. An, C. I. Clarke, B. Clausse, C. E. Clayton, J. P. Delahaye, J. Frederico, S. Gessner, S. Z. Green, M. J. Hogan, C. Joshi, M. Litos, W. Lu, K. A. Marsh, W. B. Mori, N. Vafaei-Najafabadi, D. Walz, V. Yakimenko

**Affiliations:** 1 LOA, ENSTA ParisTech, CNRS, École Polytechnique, Université Paris-Saclay, Palaiseau 91762, France; 2 Department of Physics, University of Oslo, Oslo 0316, Norway; 3 SLAC National Accelerator Laboratory, Menlo Park, California 94025, USA; 4 Department of Electrical Engineering, University of California Los Angeles, Los Angeles, California 90095, USA; 5 Department of Physics and Astronomy, University of California Los Angeles, Los Angeles, California 90095, USA; 6 IFSA Collaborative Innovation Center, Department of Engineering Physics, Tsinghua University, Beijing 10084, China

## Abstract

Plasma accelerators driven by particle beams are a very promising future accelerator technology as they can sustain high accelerating fields over long distances with high energy efficiency. They rely on the excitation of a plasma wave in the wake of a drive beam. To generate the plasma, a neutral gas can be field-ionized by the head of the drive beam, in which case the distance of acceleration and energy gain can be strongly limited by head erosion. Here we overcome this limit and demonstrate that electrons in the tail of a drive beam can be accelerated by up to 27 GeV in a high-ionization-potential gas (argon), boosting their initial 20.35 GeV energy by 130%. Particle-in-cell simulations show that the argon plasma is sustaining very high electric fields, of ∼150 GV m^−1^, over ∼20 cm. The results open new possibilities for the design of particle beam drivers and plasma sources.

## Abstract (web summary)

Plasma accelerators driven by particle beams are a promising technology, but the acceleration distance and energy gain are strongly limited by head erosion in a high-ionization-potential gas. Here the authors observe up to 130% energy boost in a self-focused electron beam, with limited head erosion.

## Graphical abstract

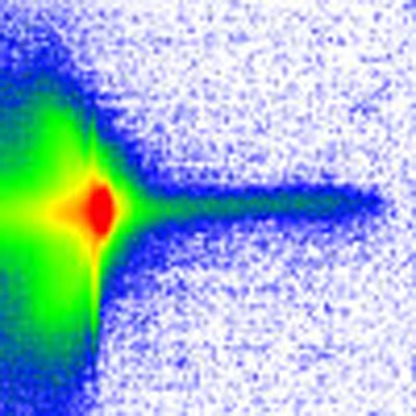

The use of plasmas for accelerating charged particles has received considerable interest in the past decades^1^,^2^,^3^,^4^,^5^,^6^,^7^,^8^,^9^. Plasmas can sustain electric fields thousands of times greater than the breakdown electric field threshold of conventional radio-frequency accelerators, paving the way towards more compact and affordable accelerators for high-energy physics at the teraelectronvolt scale. A high-amplitude plasma wave can be driven either by an intense particle or laser beam. The use of a particle beam driver (the so-called Plasma Wakefield Accelerator scheme) presents crucial advantages for future high-energy accelerator technology. In particular, it promises very competitive energy efficiency from the wall plug to the accelerated beam^10^, as well as a long interaction distance per acceleration stage because of the absence of dephasing between the particles and the plasma wave. Particle drivers are therefore attractive for any applications where energy efficiency is critical, such as linear colliders.

A particle-beam-driven plasma accelerator can be realized by focusing a short duration and dense electron beam into a stationary gas. In the blowout and self-ionized regime, the intense drive electron bunch and its Coulomb field can ionize the gas and expel all the electrons of the suddenly formed plasma from the axis of propagation, thus forming an electron-free cavity in its wake. The fields in this cavity have ideal properties^11^,^12^,^13^ for an accelerator: the transverse focusing fields are linear in radius, allowing emittance preservation, and the longitudinal accelerating field can be made constant over the length of a trailing bunch by appropriately loading the wake^14^.

The self-ionization of the gas by the leading particles of the driver presents numerous advantages^15^,^16^. It allows the production of long, uniform and high-density plasmas with no alignment or timing issues. However, this self-ionization scheme leads to a rather fast erosion of the head of the drive bunch, which can limit the acceleration length. The head erosion rate *v*_he_ was previously found to be strongly dependent on the gas ionization potential, 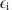, and it was shown that for electron beams that are matched to the plasma, 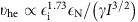, where 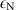 is the normalized emittance of the beam, *γ* is the Lorentz relativistic factor and *I* is the beam current at the ionization front^17^. A beam is said to be matched to the plasma when there is no oscillation of its beam size during propagation in the plasma, whereas a mismatched beam can have large envelope oscillations. Because of the difficulty to ionize species with a large 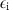 and because of the dependence of *v*_he_ in 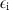, complex plasma sources, based on alkali metal vapours (for example, Rb and Li, with 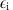
=4.2 and 5.4 eV, respectively, for the ionization of their first electron), have been developed in the past decades to be used in plasma wakefield acceleration experiments. In this particular context, noble gases (such as argon) are considered as high-ionization-potential species. In the experiment reported here, where a mismatched beam propagates in an argon gas, the conditions are expected to be strongly unfavourable for plasma acceleration because of the higher erosion rate in a high-ionization-potential gas (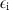
=15.8 eV for argon) and because of the emittance growth resulting from the large mismatch, and it was anticipated that such high-ionization-potential plasma accelerators would produce small energy gains (see Supplementary Discussion for details).

In this article, we explore the beam–plasma interaction within a high-ionization-potential self-ionized argon gas. We report the experimental demonstration of large energy gains in the interaction of the fully compressed electron bunch with the high-density argon plasma, boosting the initial 20.35 GeV energy of trailing electrons by up to 130%, reaching 47 GeV. The high ionization potential of the first electron of argon has implications on the physics of head erosion, as well as on the onset of gas ionization and on the dynamics of the electron beam. QuickPIC particle-in-cell simulations^18^,^19^ show that the interaction can start when the electron bunch only partially ionizes the gas, and continues with a rapid self-focusing process^20^ that leads to the ionization of all argon atoms and to the blowout regime. The simulations provide evidence for the role of the electron beam self-focusing in making the beam denser and able to drive nonlinear wakes at very high density. They also support the conclusion that long interaction distances are achieved when focusing the electron beam with large diffraction lengths, despite the anticipated high head erosion rate. The results provide a more complete understanding of the beam–plasma interaction, which will guide future optimizations of plasma acceleration stages.

## Results

### Experimental observations

**Figure 1:**
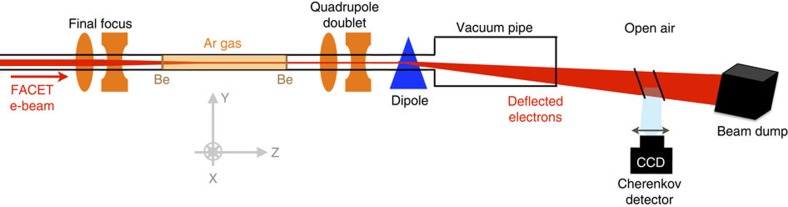
Schematic diagram of the experimental setup. The FACET electron beam is focused by final focus quadrupoles inside a volume filled with argon gas and contained by two beryllium windows. After interacting with the plasma, electrons are transported by a quadrupole doublet towards the beam dump and deflected vertically by a dipole magnet. A Cherenkov detector is used to measure the electron energy spectrum (see the Methods for details).

The schematic layout of the experimental setup is presented in Fig. 1. The electron bunch from the Facility for Advanced Accelerator Experimental Tests^16^ (FACET) at SLAC National Accelerator Laboratory, with 3.2 nC of charge, 20.35 GeV energy and a root-mean-square (r.m.s.) bunch length of ∼20 μm, was focused by the final focus quadrupoles to ∼20 μm r.m.s. beam sizes in *x* and *y*. The waist was located inside a volume filled with room temperature argon gas at pressures ranging from 2 to 32 Torr. The gas volume was contained by two beryllium windows, outside which the beam propagated in vacuum. The gas was only ionized near the waist location, and most of the gas volume was not used for acceleration. After the beam–plasma interaction, the electron beam energy spectrum was measured with a focusing-imaging spectrometer (see the Methods for details).

**Figure 2:**
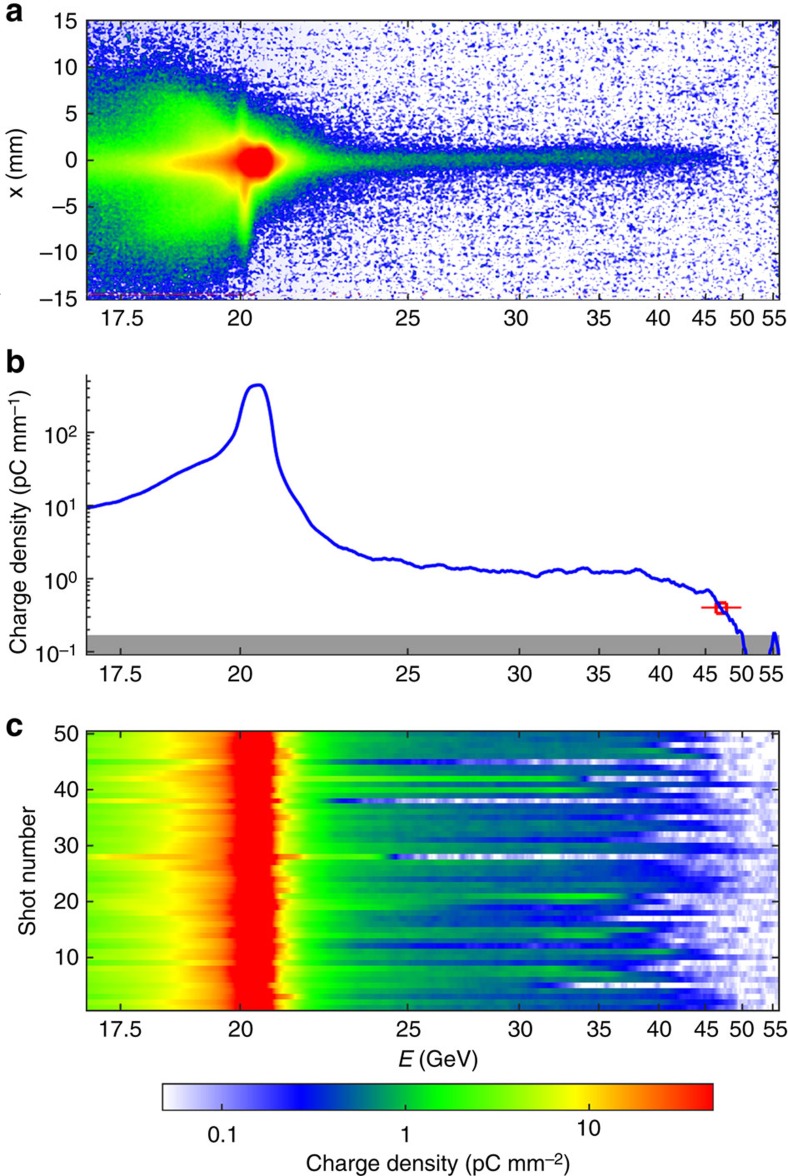
Electron energy spectra. (**a**) Single shot image of the electron energy spectrum measured by the Cherenkov detector (see the Methods for details). (**b**) Projection of the image (**a**) on the electron energy axis, showing a maximum energy at 47±2.5 GeV. The horizontal error bar accounts for resolution limit due to finite beam size and for error due to pointing fluctuations, and the grey area indicates the detection threshold. (**c**) Waterfall plot showing the peak charge density as a function of electron energy for 50 consecutive electron bunches.

Figure 2 presents the measured energy spectra of the electron beam after traversing the plasma when the argon pressure was set to 20 Torr, corresponding to a neutral atom density of 6.5 × 10^17^ cm^−3^. For the bunch shown in Fig. 2a,b, electrons were observed to be accelerated up to a maximum energy of 47±2.5 GeV, corresponding to an energy gain of ≃27 GeV and an energy boost of ≃130%. By measuring the beam size in *x* at the detector location and using the transport matrix from the plasma exit to the detector, the divergence of these accelerated electrons at the exit of the plasma was deduced to be extremely small, less than ≃75 μrad (r.m.s.). The charge of the accelerated electrons (with energies higher than 23 GeV) was ∼40 pC. Fifty consecutive shots with the same experimental conditions are shown in Fig. 2c, demonstrating that these large energy gains were observed frequently. More precisely, in this data set, 24% of the shots had a maximum energy greater than 45 GeV, 68% were over 40 GeV and 94% over 30 GeV. The average maximum energy gain was 41.3±0.8 (stat.) GeV. In the experiment, different beam parameters (bunch length, shape of the longitudinal profile, beam size and emittance) fluctuated from one shot to the next and affected the experimental outcome, and lead to the observed shot-to-shot fluctuations.

**Figure 3:**
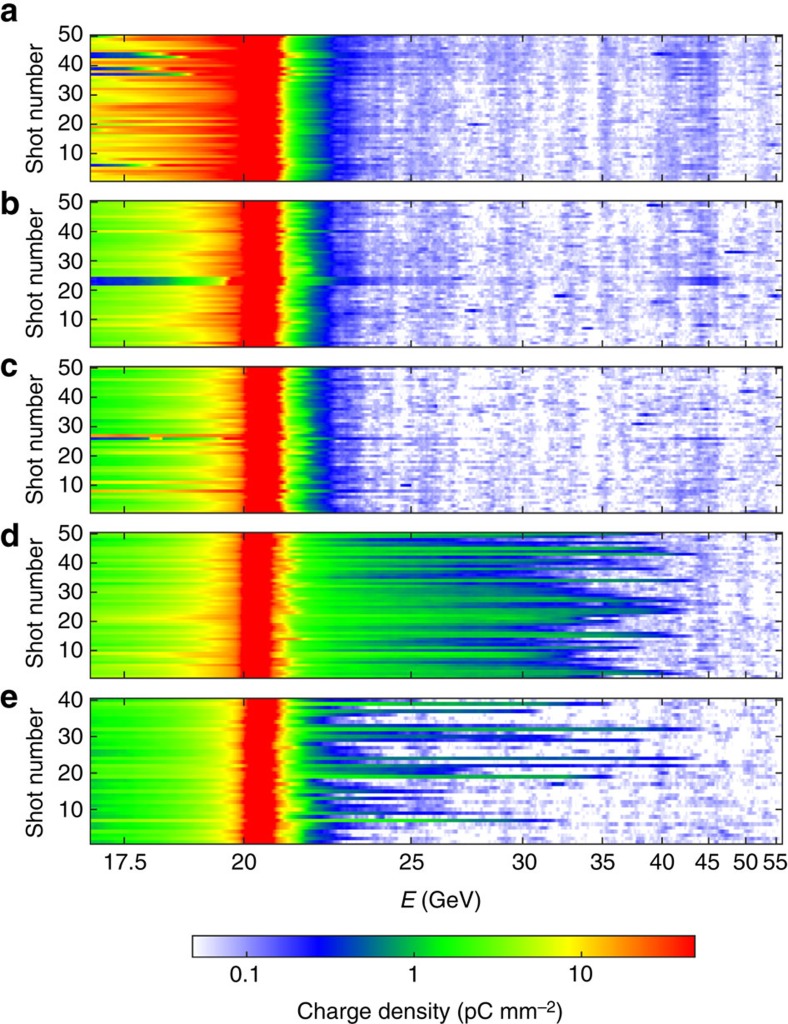
Energy gain as a function of argon pressure. Waterfall plots of peak charge density as a function of electron energy for an argon pressure of 2 Torr (**a**), 4 Torr (**b**), 8 Torr (**c**), 16 Torr (**d**) and 32 Torr (**e**). Each waterfall plot shows 50 consecutive shots taken under the same experimental conditions (except for **e**, where only 40 shots are presented).

Electron acceleration was found to be strongly dependent upon the argon pressure, that is, on the plasma density. In the experiment, no acceleration was observed at pressures of 2, 4 and 8 Torr (see Fig. 3a–c for details). Large energy gains, typically doubling the initial electron energy, first appear when increasing the pressure to 16 Torr (see Fig. 3d for details), which corresponds to a neutral atom density of 5.2 × 10^17^ cm^−3^. Strong acceleration was still present at the highest pressure of the scan, 32 Torr, although the interaction was less stable (see Fig. 3e for details). The maximum energy loss is another important physical quantity characterizing the strength of the beam–plasma interaction. At 2 Torr, the maximum energy loss was fluctuating between 4 GeV and more than 9 GeV, with a median value of 7 GeV. For all other pressures, the maximum energy loss was observed to be always greater than 9 GeV (limited by the camera field of view).

### Numerical simulations and interpretation

**Figure 4:**
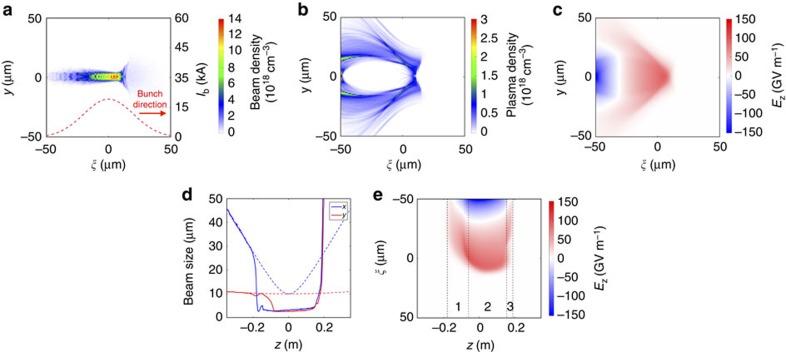
QuickPIC simulation at *P*=16 Torr. Simulated beam electron density (**a**), plasma electron density (**b**) and longitudinal electric field *E*_*z*_ (**c**) at *z*=0, the location where the beam reaches its minimum transverse size in vacuum. (**d**) Evolution of the *x* and *y* beam sizes (r.m.s. values with 10% cut) of a slice of the bunch located at *ξ*=*z*−*ct*=−10 μm for propagation through a plasma (solid lines) and for propagation in vacuum (dashed lines). This slice is inside the blowout region when plasma is present. (**e**) Evolution of the longitudinal on-axis electric field *E*_*z*_ during beam propagation through the argon gas volume. The three different regions are described in the text.

To better understand the experimental observation of large energy gains in high-density and high-ionization-potential plasma accelerators, particle-in-cell simulations of the interaction between the electron beam and the argon gas were performed using the three-dimensional quasi-static code QuickPIC^18^,^19^ (see the Methods for details). As the interaction can only start when the beam is small enough to ionize, it is not necessary to simulate the propagation through the entire gas volume, but only through the region where ionization can occur. Experimentally, the beam enters the argon volume long before reaching its minimum transverse size. To reproduce this condition, the simulation starts at a point where the beam size is sufficiently large to ensure no self-ionization occurs, and the gas density is constant along the propagation axis. Figure 4a–c shows the simulated electron beam density, the plasma electron density and the longitudinal field of the wakefield at *z*=0, the location of the vacuum beam waist, for an argon pressure of 16 Torr or, equivalently, a neutral atom density of 5.2 × 10^17^ cm^−3^.

To ionize 1% of the argon atoms in a time Δ*t*=10 fs (equivalent to Δ*ξ*=3 μm), the required electric field is 29 GV m^−1^. With a beam peak electric field of 52 GV m^−1^ (at the vacuum beam waist), it is thus only near the middle of the bunch, where the current and the Coulomb electric field are the highest, that the beam is able to begin to ionize the argon gas. Simulations shown in Fig. 4a–c illustrate that the portion of the bunch ahead of the ionization front propagates as in vacuum. The transverse beam size behind the ionization front is observed to be extremely small in comparison. This can be explained by a strong self-focusing of the electron beam^20^ as it starts interacting with the plasma.

The evolution of the beam sizes (along *x* and *y*) of a slice of the bunch located behind the ionization front during beam propagation through the argon volume is represented in Fig. 4d. When the beam starts ionizing a small fraction of the argon gas, a wake is excited that focuses the rest of the beam to a smaller transverse size. As the beam density increases the ionization fraction, the wakefield amplitude and the focusing force increase, leading to an even faster reduction of the beam size. This self-focusing process continues in a positive feedback loop until all argon atoms are ionized and a full blowout wake regime is reached, or until the emittance term balances the focusing force in the beam envelope equation^21^. We note that the feedback mechanism associated to self-focusing leads to a very different behaviour compared with the transverse dynamics of a beam in the blowout regime (no feedback), in which case betatron oscillations of the beam envelope are observed^22^ (see Supplementary Fig. 1 for details).

Electron beam self-focusing leads to a strong increase in the beam density, up to ∼10^19^ cm^−3^. This allows the beam to easily reach the blowout regime (*n*_beam_≫*n*_plasma_) in high-density plasmas (see Fig. 4b for details), with plasma electron densities above 10^18^ cm^−3^, and thus to generate very high electric fields, of the order of 150 GV m^−1^ (see Fig. 4c for details).

To generate a large net energy gain, this high electric field must be sustained over a long distance. Figure 4e shows the evolution of the simulated on-axis longitudinal electric field *E*_*z*_ during beam propagation through the argon volume. The high electric field is maintained over a distance of about 20 cm. In this simulation, the beta function at the focus β* or vacuum diffraction length, analogous to the Rayleigh length of diffracting light beams, is much larger than the required beta function for matching (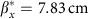 and 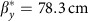, whereas 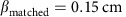 in a Ar^2+^ plasma at 16 Torr). For this simulation of an initially unmatched beam, the dynamics of head erosion are very different from the idealized head erosion model for a matched beam^17^, which predicts a continuous head erosion. At first, the wake has a superluminal phase velocity during the self-focusing process (region 1 in Fig. 4e). This arises from the dependence of the wakefields on the *ξ*=*z*−*ct* variable (the variable comoving at the speed of light in the direction of motion): slices at the rear of the bunch are subjected to a stronger focusing force than those at the front, and therefore they are self-focused first. This leads to a wake that moves from the rear of the bunch to the front (see Supplementary Movie 1 for details), with a superluminal phase velocity. After the self-focusing process, the beam undergoes almost no head erosion for ∼20 cm of propagation (region 2). In this stage, the head of the bunch that initiates ionization evolves nearly as in vacuum and provides an onset of ionization sufficient to provide guidance to the remaining part of the beam, in a pseudo-steady-state regime^17^ and for a distance of the order of its beta function. When a part of the bunch has been fully decelerated (end of region 2), the wake front suddenly recedes towards the rear of the bunch, and a wake is driven for a very short period of time (region 3), after which the beam-plasma interaction is terminated.

One generally favours the use of short beta functions to match the drive beam to the plasma and avoid possible emittance growth usually leading to fast head erosion. However, our results show that one can also use long beta functions, of the order or greater than the desired plasma stage length. The use of large beta functions from softer focusing thus permits a long interaction distance. The long interaction distance together with the high electric fields produced in high-density plasmas, lead to energy gains of 20–30 GeV as observed in the experiment. In this regime, the interaction distance may thus reach the depletion length (where a significant number of particles have been decelerated to near their rest energy). This is a requirement for a high-efficiency accelerator where most of the energy of the drive beam is transferred to the wake.

**Figure 5:**
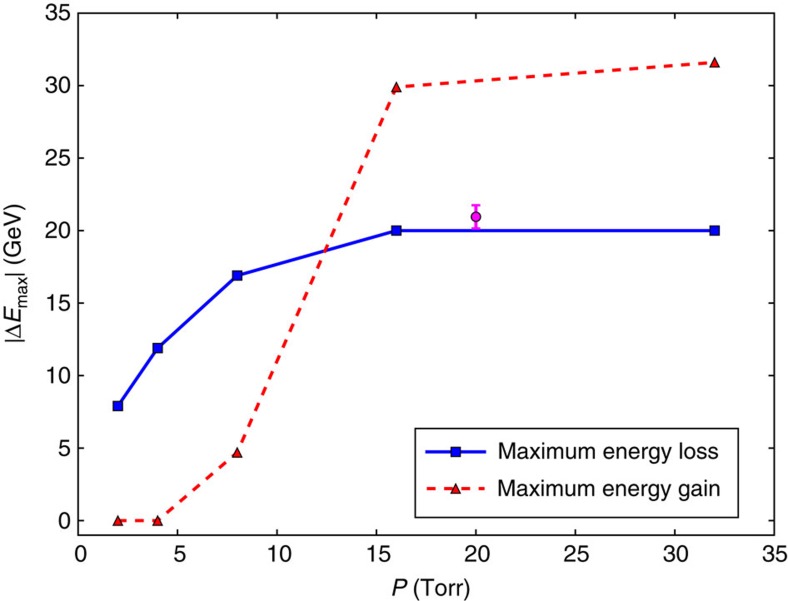
Energy gain and loss in simulations. The red triangles (blue squares) represent the maximum energy gain (maximum energy loss) observed at the end of the beam-plasma interaction in QuickPIC simulations (see the Methods for details) for different argon pressures. The magenta circle and its error bar represent the experimental average maximum energy gain corresponding to the data of Fig. 2 and its s.e.m.

Figure 5 shows the energy gain and loss observed in QuickPIC simulations as a function of the argon pressure *P*. The energy loss increases with *P* as the result of the increase in the *E*-field amplitude, until it reaches its maximum value of 20 GeV corresponding to full deceleration of a part of the beam. The energy losses in the simulations are compatible with the experimental measurements, which show a typical energy loss of 7 GeV at 2 Torr and energy losses always greater than 9 GeV for pressures above 4 Torr. As in the experiment, no energy gain is observed in the simulations at small pressures, where the blowout cavity is too long compared with the bunch length, and no beam particles are probing the accelerating phase of the wake. At 16 and 32 Torr, some trailing electrons are accelerated to very high energies, reaching 50 GeV, that is, an energy gain of 30 GeV. The experimental results are in very good agreement with these simulations.

## Discussion

Plasma acceleration with high energy gains, up to 27 GeV, was demonstrated in a self-ionized, high-density and high-ionization-potential argon gas. The initial 20.35 GeV electron energy was boosted by up to ∼130%. The result can be explained through the process of electron beam self-focusing, which allows the beam to drive nonlinear wakes in high-density plasmas, thereby generating very high electric fields, of the order of 150 GV m^−1^. Furthermore, by using large beta functions relative to the matched values, we entered a different regime of head erosion that can provide a significant interaction length in high-ionization-potential plasma accelerators. For our experimental conditions, simulations indicate that the interaction length was of the order of 20 cm and that the energy of a portion of the beam was fully depleted.

The physics insight provided by the observation of large energy gains in a noble gas plasma source opens new possibilities for the design of optimal and simpler particle beam drivers as well as the design of plasma sources. In addition, a plasma target made from a room temperature gas is relatively simple and easily scalable over a wide range of densities and lengths, can be operated at high repetition rates, and can provide better longitudinal density uniformity than lower ionization-potential alkali metal vapours from heat pipe ovens. Finally, the electron beam self-focusing process opens new perspectives for applications where very small beam sizes are required (for example, luminosity enhancement for final focus systems, nanoscale wigglers^23^ and so on).

## Methods

### Electron beam parameters and target

The FACET electron beam had an energy of 20.35±0.05 GeV, a charge of 3.2±0.1 nC and a bunch length of 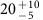 μm (r.m.s.). It was focused down to beam sizes in *x* and *y* of 20±10 μm (r.m.s.). The final focus configuration used beta functions at the focus of either 10 cm (in *x*) by 10 cm (in *y*; Fig. 2 data), or 12 cm (in *x*) by 100 cm (in *y*; Fig. 3 data). The beam normalized transverse emittance was ∼80±2 mm.mrad (in *x*) by 25±2 mm.mrad (in *y*) before final compression and the final focus. The peak current of the beam was *I*_peak_=19±7 kA. The beam was focused inside a 3.41-m-long volume filled with stationary argon gas at pressures ranging from 2 to 32 Torr. This 3.41-m-long gas volume was contained by two Be windows (Fig. 1), outside of which the beam propagated in vacuum. The beam waist was located either 1.16 m (Fig. 2 data) or 0.59 m (Fig. 3 data) downstream of the first Be window, guaranteeing a large enough beam size at the window to prevent damaging it. The first Be window induced multiple scattering to the beam particles, with a r.m.s. scattering angle of 5.3 mrad, and contributed to the beam size indicated above.

### Cherenkov electron spectrometer

The electrons are deflected vertically by a dipole magnet (Fig. 1) whose equivalent length and *B* field are, respectively, 0.978 m and 0.42 T. Electrons of 20.35 GeV energy experience a 5.73-mrad deflection. At the location of the detector, the electron vertical position provides a measurement of the electron energy. A quadrupole doublet is used to image the expected plasma exit onto the detector. The imaging conditions only hold for the nominal 20.35 GeV energy, and the beam is defocused at other energies. The energy resolution depends on the finite vertical betatron beam size at the detector and an energy error arises from pointing fluctuations. The detector consists of two 12-bit visible charge-coupled device cameras imaging the Cherenkov light produced by electrons as they pass through a 5-cm-thick air gap, limited by two silicon wafers positioned at an angle of 45° to the beam. The first camera images the energy range from 17 to 60 GeV, whereas the second camera images the energy range from 11.5 to 23 GeV.

### Particle-in-cell simulations

The three-dimensional quasi-static code QuickPIC^18^,^19^ was used to simulate the interaction between the FACET electron beam and the argon gas. The simulation mesh consists of 512 (in *x*) × 512 (in *y*) × 256 (in *ξ*=*z*−*ct*) cells. The cell size is 0.742 μm (in *x*) × 0.742 μm (in *y*) × 0.453 μm (in *ξ*=*z*−*ct*). The simulation window moves at the speed of light in beam direction, along the *z* axis. Positive ions, once generated, are considered in the simulations to be stationary. In the quasi-static approximation, the plasma response time scale and the beam evolution time scale are separated. The plasma response is first computed for a non-evolving electron beam, and then the beam is advanced according to the plasma forces. The number of beam macro-particles is 4.2 × 10^6^ and the number of plasma macro-particles is 4.2 × 10^6^. The beam evolution step size is 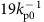, where 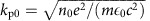 and *n*_0_ is the neutral atom density of the argon gas.

The simulation begins at *z*=−0.35 m, where *z*=0 is the waist location, and the electron beam is initialized such that it would focus to a 10 μm (in *x*) by 10 μm (in *y*) r.m.s. spot size in vacuum, with 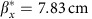 and 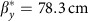. The beam has an energy of 20 GeV, a bunch length of 20 μm (r.m.s.), and contains 2 × 10^10^ electrons. The beam propagates in a constant density argon gas (whose neutral atom density is chosen depending on the desired argon pressure), and the code describes the field ionization of the first two electrons of argon. The beam size of a slice (shown in Fig. 4d) is calculated by taking the projection of the beam density along the *x* (or *y*) axis, cutting the projection at 10% of its maximum and computing the r.m.s. value of the cut projection. A movie of the 16-Torr simulation is available as Supplementary Movie 1. The simulations were carried out on the Hoffman 2 Cluster at University of California, Los Angeles.

### Data availability

The data that support the findings of this study are available from the corresponding author upon request.

## Additional information

**How to cite this article:** Corde, S. *et al.* High-field plasma acceleration in a high-ionization-potential gas. *Nat. Commun.* 7:11898 doi: 10.1038/ncomms11898 (2016).

## Supplementary Material

Supplementary Information Supplementary Figures 1-2, Supplementary Discussion and Supplementary References

Supplementary Movie 1 Simulation video depicting the evolution of the plasma wake and the electron bunch as it propagates in neutral argon gas
