# Supplementary material for: High-field plasma acceleration in a high-ionization-potential gas
Source: Nat Commun. 2016 Jun 17;7:11898. doi: 10.1038/ncomms11898 (PMC4915014; doi:10.1038/ncomms11898)
Supplement: Supplementary Information — Supplementary Figures 1-2, Supplementary Discussion and Supplementary References [file ncomms11898-s1.pdf]

SUPPLEMENTARY INFORMATION

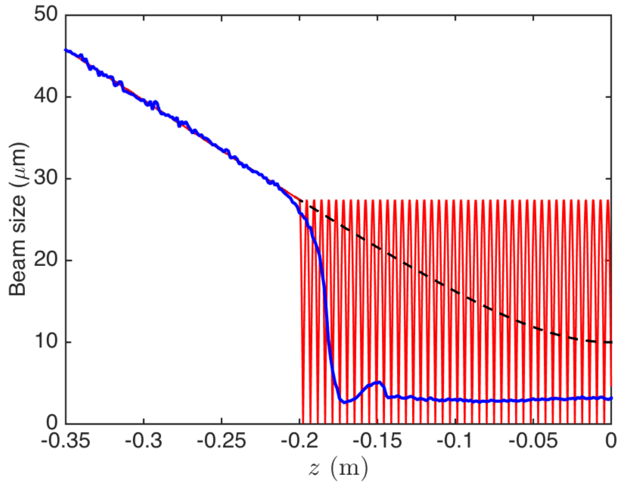

Supplementary Figure 1 | Comparison between self-focusing and betatron oscillations. Evolution of the beam size for a beam propagating in vacuum (black dashed line), for a self-focused beam (blue solid line) with a feedback between the beam and the transverse wakefield (curve identical to the evolution of the *x* beam size in Fig. 4(d)), and for a beam entering a 16 Torr Ar^{2+} plasma in the blowout regime at z = -0.2 m (red solid line) and experiencing a linear transverse force (no feedback), as previously studied in Ref. [1]. In the last case, the beam envelope oscillates at half the betatron wavelength of beam particles (λ_β). In the self-focused case, the feedback process allows the beam to reach and maintain a very small transverse size.

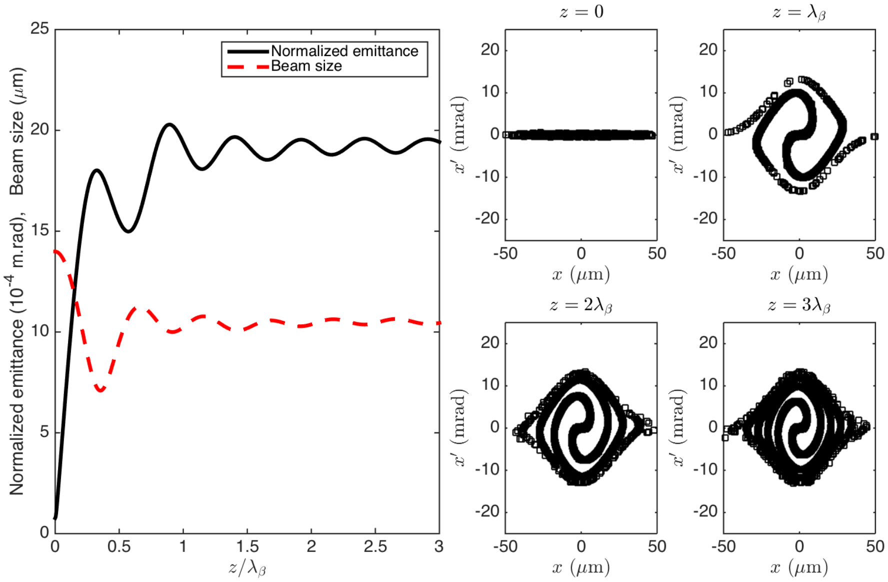

Supplementary Figure 2 | Example of emittance growth of a mismatched beam in a nonlinear transverse force. The transverse force is given by a linear force modulated by a gaussian envelope: $F_x = -0.5\ m\omega_p^2\, x \times \exp(-x^2/(2\sigma_x^2))$, where σ_x is the initial r.m.s. beam size and ω_p is the plasma frequency of a 16 Torr Ar^{2+} plasma. The beam is initiated with σ_x = 14 µm and β_x = 0.1 m, and with an energy of 20.35 GeV. The evolution of the normalized emittance and beam size are shown on the left, and the beam trace space (x,x') at $z = 0$, $z = \lambda_\beta$, $z = 2\lambda_\beta$, and $z = 3\lambda_\beta$ are shown on the right (λ_β is the betatron wavelength).

Supplementary Discussion

The experiment reported in this article uses a mismatched beam in a high-ionization-potential gas (argon, whose ionization potential is 15.8 eV for the first electron). These conditions are expected to be strongly unfavourable because of the higher head erosion rate in the argon gas and because of the emittance growth due to the large mismatch.

The theory for head erosion in Ref. [2] is developed for the case of a matched beam, and the effect of a mismatch is also discussed. Matched beams are considered as the optimal drivers because if the beam is mismatched, its emittance will grow as the beam evolve towards a matched state, thus resulting in a higher head erosion rate. In our case, the growth of the mismatched beam emittance is driven by the nonlinear transverse force that is present in the region where the wake is forming and where head erosion is taking place. The typical length scale for the emittance growth in this case is the betatron period (see Supplementary Figure 2), which is λ_β = 9.2 mm for a Ar^{2+} plasma at 16 Torr. Once the emittance has grown, the beam is matched and the erosion rate can be estimated. Using our parameters and considering a beam size of 10 μm after emittance growth, one finds $v_{HE} = 2.9 \times 10^3$ μm/m at the peak of the current profile, and by integrating over the current profile, one obtains a length of L_{HE} = 14 mm for head erosion. From these considerations, the expectation for the interaction length, with a mismatched beam in a high-ionization potential gas, is in the centimeter range, which suggests that only very small energy gains should be observed.

Supplementary References

[1] C. E. Clayton *et al.*, *Transverse Envelope Dynamics of a 28.5- GeV Electron Beam in a Long Plasma*, Phys. Rev. Lett. 88, 154801 (2002).

[2] I. Blumenfeld, *Scaling of the longitudinal electric fields and transformer ratio in a non-linear plasma wakefield accelerator*, Ph.D. thesis (Stanford University, 2009), Chapter 3.
